# A Terrible Sore Throat: Pharyngitis in an Emergency Department Patient With a Final Diagnosis of Acute Myelogenous Leukemia

**DOI:** 10.7759/cureus.23306

**Published:** 2022-03-18

**Authors:** Susannah Boulet, Melody L Milliron, Christopher Karns

**Affiliations:** 1 Emergency Medicine, Allegheny Health Network, Saint Vincent Hospital, Erie, USA

**Keywords:** hematology-oncology, clinical gestalt, acute myelogenous leukemia, sore throat, acute pharyngitis

## Abstract

Acute pharyngitis is a common complaint in emergency department (ED), urgent care, and primary care settings. Most cases are due to bacterial or viral infections easily treated with antibiotics or supportive care. However, serious pathologies in the pharyngitis differential include Ludwig’s angina, peritonsillar abscess, retropharyngeal abscess, and bacterial tracheitis. Rarely, oncologic conditions such as leukemia may initially present as pharyngitis in an acute care setting. We present a case of pharyngitis in a 32-year-old male ED patient with a final diagnosis of acute myelogenous leukemia (AML). Knowledge of the appropriate ED evaluation of AML is key for accurate diagnosis and prompt referral to avoid unnecessary patient morbidity and mortality.

## Introduction

Acute myeloid leukemia (AML) is a type of blood cancer. Specifically, it is a cancer of myeloid cells, a white blood cell lineage arising from bone marrow [1]. During hematopoiesis, stem cells residing in the bone marrow differentiate. These hematopoietic stem cells (HSCs) can differentiate into either a common myeloid progenitor cell or a common lymphoid progenitor cell. The common myeloid progenitor cells can further differentiate into myeloblasts, which are destined to end up as basophils, neutrophils, eosinophils, and monocytes [2].

In AML, a mutation in the myeloblast cell causes halting in the maturation process [3]. These immature cells accumulate, and apoptosis is halted, thereby overcrowding the bone marrow with unproductive cells [3,4]. This overcrowding interferes with the production of other normal blood cells and leads to bone marrow failure with findings of neutropenia, anemia, and thrombocytopenia. Thus, patients may present with bleeding, infection, or generalized fatigue. While a comprehensive physical exam may show characteristics of this disease, diagnosis is obtained with a complete blood count consisting of at least 20% blasts on peripheral blood smear [3].

## Case presentation

A 32-year-old male presented to the emergency department (ED) with a chief complaint of a sore throat. He stated his symptoms began three days prior with associated fever, generalized fatigue and “nodes” in his anterior neck. He denied any difficulty swallowing, cough, or dysphonia. He admitted to taking amoxicillin for three days that was “left over” from a prior prescription. The patient stated his brother was recently diagnosed with “strep throat.” He denied any pertinent past medical, social, or surgical history.

Initial vital signs included a respiratory rate of 16 breaths per minute, oxygenating at 100% oxygen saturation (SpO2) on room air, and afebrile. He was hypertensive with an initial blood pressure reading of 158/106 mmHg and tachycardic at a rate of 104 beats per minute. Initial exam yielded a well-appearing man, airway intact, bilateral breath sounds and strong peripheral pulses. His posterior pharynx was erythematous but no notable peritonsillar abscess or exudates were seen. He had no dysphonia, no trismus, and no tongue elevation. Palpation of the patient’s neck was remarkable only for subcentimeter areas of anterior lymphadenopathy bilaterally. Cardiovascular, pulmonary, and abdominal exams were benign.

Upon initial evaluation, the treating physician’s clinical gestalt led to orders including a rapid strep test and Monospot test, both of which were negative, and a throat culture as additional testing was pursued. His basic metabolic panel revealed an elevated creatinine at 1.7. Computerized tomography of the neck without intravenous contrast was negative for acute findings. The lab then called with critical results after a manual peripheral smear was performed. The patient’s complete blood count, revealed significantly elevated white blood cells at 172,000 with 31% blasts, leading to his final ED diagnosis.

The patient was diagnosed with AML, pharyngitis, and acute renal insufficiency. Infectious disease was consulted for antibiotic coverage recommendations while patient was awaiting transfer to another facility for higher level of hematology/oncology care. Infectious disease recommended broad-spectrum antibiotic administration with ceftriaxone. Patient had no central nervous system changes or cardiovascular manifestations of hyperviscosity. However, patient’s renal dysfunction was of concern as a manifestation of suspected life-threatening blast crisis, and patient was transferred to another facility for higher level of hematology/oncology care for initiation of remission induction therapy. Unfortunately, the patient expired four months later due to AML complications.

## Discussion

Acute pharyngitis is a common complaint in ED, urgent care, and primary care settings. Most cases are due to bacterial or viral infections easily treated with antibiotics or supportive care. However, serious pathologies in the pharyngitis differential include Ludwig’s angina, peritonsillar abscess, retropharyngeal abscess and bacterial tracheitis. Rarely, oncologic entities such as AML may initially present as pharyngitis in an acute care setting.

Acute myeloid leukemia is a hematologic malignancy of myeloid blasts. It is the most common type of acute leukemia in adults [1]. There are over 20,000 new cases of AML per year diagnosed in the United States (US) [3]. This form of leukemia results in the most deaths per year in the US [1]. The average age at diagnosis is 65 years old and males have a 5:3 predominance over females [3].

Due to this mutation in signal transduction, myeloid blasts expand in the bone marrow at a developmentally immature stage. This expansion of “blasts” hinders growth of normal blood cells resulting in neutropenia, anemia, thrombocytopenia, and ultimately bone marrow failure. Patients may present with a wide range of symptoms owing to their ineffective differentiated cell production. Anemia can result in fatigue, dyspnea, and chest pain. Neutropenia can result in increased or recurrent infections, fevers, or malaise. Lastly, thrombocytopenia can present as easy bruising, petechiae, joint or bone pain [3].

Differential diagnosis of a patient presenting with anemia, neutropenia, or thrombocytopenia may include leukemias such as acute lymphoblastic leukemia (ALL), chronic myelogenous leukemia (CML), aplastic anemia, B-cell lymphoma, or myelodysplastic syndrome (MDS) [3]. These diagnoses carry additional lab findings, but the diagnostic criteria of AML according to 2016 WHO guidelines are greater than or equal to 20% blast cells on peripheral blood or bone marrow [1]. Other tests to include in the ED evaluation and to aid in treatment are coagulation studies, comprehensive metabolic panel, chest radiograph and ECG. Evaluation of lactate dehydrogenase (LDH) and uric acid levels are important to rule out tumor lysis syndrome, which is a medical emergency [3].

The prognosis for acute myeloid leukemia is variable and resides on factors such as the type of chromosomal abnormalities, gene mutations, age of patient, and white blood cell count [3]. For example, genetic mutations such as TP53, KIT, and FLT3-ITD all exhibit a different prognosis and genetic testing should be performed to help guide pharmacologic therapy [1]. Regardless of therapeutic interventions, the prognosis remains poor for the elderly population [3]. ED diagnosis can lead to earlier diagnosis, interventions and potentially a more favorable outcome.

## Conclusions

While a simple case of pharyngitis is more probable, it is important to consider other life-threatening pathologic causes in the differential. Keeping a broad differential diagnosis, even with a common complaint of pharyngitis and pursuing further testing when indicated can result in a more rapid diagnosis. Although this patient unfortunately expired, prompt recognition and diagnosis of AML in the ED can potentially lead to rapid interventions which may improve the patient’s morbidity and mortality.
